# How Older People Experience the Age-Friendliness of Their City: Development of the Age-Friendly Cities and Communities Questionnaire

**DOI:** 10.3390/ijerph17186867

**Published:** 2020-09-20

**Authors:** Jeroen Dikken, Rudy F.M. van den Hoven, Willeke H. van Staalduinen, Loes M.T. Hulsebosch-Janssen, Joost van Hoof

**Affiliations:** 1 Chair of Urban Ageing, Faculty of Social Work & Education, The Hague University of Applied Sciences, Johanna Westerdijkplein 75, 2521 EN Den Haag, The Netherlands; J.Dikken@hhs.nl (J.D.); r.f.m.vandenhoven@hhs.nl (R.F.M.v.d.H.); 2 Faculty of Health, Nutrition & Sport, The Hague University of Applied Sciences, Johanna Westerdijkplein 75, 2521 EN Den Haag, The Netherlands; 3 AFEdemy—Academy on age-friendly environments in Europe, Buurtje 2, 2802 BE Gouda, The Netherlands; willeke@afedemy.eu; 4 Hulsebosch Advies, Lissenvaart 43, 2724 SJ Zoetermeer, The Netherlands; hulsebosch@hm-advies.nl; 5 Institute of Spatial Management, Faculty of Environmental Engineering and Geodesy, Wrocław University of Environmental and Life Sciences, ul. Grunwaldzka 55, 50-357 Wrocław, Poland

**Keywords:** survey, questionnaire, validation, age-friendly, age-friendly cities, older people, age-friendliness, AFCCQ

## Abstract

The World Health Organization engages cities and communities all over the world in becoming age-friendly. There is a need for assessing the age-friendliness of cities and communities by means of a transparently constructed and validated tool which measures the construct as a whole. The aim of this study was to develop a questionnaire measuring age-friendliness, providing full transparency and reproducibility. The development and validation of the Age Friendly Cities and Communities Questionnaire (AFCCQ) followed the criteria of the COnsensus-based Standards for selection of health Measurement INstruments (COSMIN). Four phases were followed: (1) development of the conceptual model, themes and items; (2) initial (qualitative) validation; (3) psychometric validation, and (4) translating the instrument using the forward-backward translation method. This rigorous process of development and validation resulted in a valid, psychometrically sound, comprehensive 23-item questionnaire. This questionnaire can be used to measure older people’s experiences regarding the eight domains of the WHO Age-Friendly Cities model, and an additional financial domain. The AFCCQ allows practitioners and researchers to capture the age-friendliness of a city or community in a numerical fashion, which helps monitor the age-friendliness and the potential impact of policies or social programmes. The AFCCQ was created in Dutch and translated into British-English.

## 1. Introduction

For over a decade, the World Health Organization (WHO) has been involved in engaging and assisting cities and communities all over the world in becoming “age-friendly” [1,2,3,4,5,6,7,8,9,10]. The WHO proposed that policies, services, and structures in an age-friendly city, which are related to the physical and social environment, are designed to support and enable older people to “age actively”. A growing number of cities and communities worldwide are striving to better meet the needs of their older residents. The WHO Global Network for Age-Friendly Cities and Communities was established to foster the exchange of experience and mutual learning between cities and communities of different sizes worldwide [11]. According to the WHO, the efforts of these cities and communities to become more age-friendly take place within very diverse cultural and socio-economic contexts. The common ground between these network partners is “the desire and commitment to promote healthy and active ageing and a good quality of life for their older residents”. Each member monitors its progress along the age-friendly milestones, and there is a global database of age-friendly practices, as well as a library containing member-uploaded toolkits, publications, research updates and videos [12].

In 2018, the WHO signalled a number of knowledge gaps in terms of age-friendly cities and communities [12]. The largest of these gaps is that the WHO’s age-friendly cities approach needs to strengthen its focus on multisectoral action that delivers outcomes in ways that reduce inequities. In the WHO’s own words, guidance and tools are needed to support cities and communities to make decisions around which actions are most likely to ensure these outcomes and not leave any groups behind in the process of development [12] (p. 18). According to Buckner et al. [13,14], one of the challenges for the evaluation of age-friendly city initiatives is to identify an evidence-based approach that (i) can be applied in different contexts, (ii) reflects the complexity of the initiatives, (iii) draws on sound data to make assessments of effectiveness, and (iv) presents findings clearly to a mixed audience. The researchers identified ten thematic areas where evidence was required, namely: political support; leadership and governance; financial and human resources; involvement of older people; priorities based on needs assessment; application of existing frameworks for assessing age-friendliness; provision; evidence-based interventions; coordination, collaboration and interlinkages; and monitoring and evaluation.

In short, there is a great need for monitoring, evaluating, measuring and assessing the age-friendliness of cities and communities. The Checklist of Essential Features of Age-Friendly Cities [15] contains a large number of features which are essential to an age-friendly city, and was based on the results of the WHO Global Age-Friendly Cities project consultation in 33 cities in 22 countries. The checklist is a guide for a city’s self-assessment and a map for charting progress. This does not mean that all domains are equally relevant in all countries and cities, depending on the status quo in each country that wishes to evaluate. For conducting supportive research, one could also find inspiration in the set of core indicators published by the WHO in 2015 [16], as well as a list of research methodologies, which can be used to investigate the age-friendliness of a city. On top of this, Fulmer et al. [17] called for the creation of an ecosystem, where each of the age-friendly initiatives can create synergies and additional momentum as the population continues to age. Their vision for an age-friendly ecosystem encompasses the lived environment, social determinants of health, the healthcare system, and a prevention-focused public health system. At the same time, Marston and van Hoof [18] argued about the apparent lack of consideration of technology in the existing age-friendly cities literature, which is another direction of investigation that should be included in the assessment of the age-friendliness of cities and communities, and also called for a new ecosystem. To date, despite these noble calls for new ecosystems, the lack of measurability of the age-friendliness of cities and communities is a clear weakness in moving the agenda forward.

Qualitative approaches have tried to measure and assess the age-friendliness of a city, for instance, through photoproduction [19] and photovoice [20] methods, or through citizen science programs of research [21,22]. Various researchers have tried to come up with a more quantitative approach to measure the age-friendliness, often taking the Checklist of Essential Features of Age-Friendly Cities [15] as a basis for their work. Luciano et al. [23] presented a framework for the assessment of the age-appropriateness of housing through a number of metrics that detect and identify physical and non-physical features of a home environment to enable ageing in place. Their study combined data from a systematic literature review expert opinion. A total of 71 metrics were identified and divided into eight main domains to describe the framework. Their study only addressed the age-friendliness of housing, which is just one of the eight domains highlighted by the WHO. In addition, Flores et al. [24] noted a lack of empirical research exploring the impact of age-friendly cities on older people. Therefore, they evaluated an age-friendly city by analysing its relationship with life satisfaction, taking into account the age cohort variables of older people and whether they live alone or with someone else. They conducted a two-stage study, in which 66 people participated in the qualitative analysis (focus groups) in Stage I and 203 in the quantitative analysis (survey methodology) of Stage II. The regression analysis indicates that for all age cohorts, community support and health services were significantly associated with life satisfaction. Social participation and outdoor spaces and buildings were only significantly associated with life satisfaction for older people who live together. Their research did not produce a validated questionnaire. Zaman and Thornton [25] identified the priority indicators for age-friendly development at the local government level in Unley, South Australia. The study conducted a community perception survey to identify the important indicators, followed by a focus group consultation to identify the priority indicators based on local settings. The study identified 25 indicators as priority indicators for the City of Unley that need to be considered for the development of an age-friendly Unley. Garner and Holland [26] described the development and validation of the Age-Friendly Environment Assessment Tool (AFEAT), assessing whether individual function and frailty impact on perceptions of environmental age-friendliness. The AFEAT was developed using the WHO Age-Friendly Environment Checklist. A total of 132 participants from the United Kingdom, aged 58–96, took part. The AFEAT, which comprises ten items (using a five-point Likert scale system), assesses individual perceptions of the environment. The AFEAT showed the need for an individual-oriented age-friendly environment tool.

All of the instruments described lack transparency of (several phases of) the development and validation process, they do not measure the age-friendliness construct as a whole (covering all eight domains as defined by the WHO) and the methodological rigour in the development process can be questioned, influencing the reliability, validity and usability of these instruments. Therefore, the aim of this study was to develop a questionnaire measuring the age-friendliness of a city or community, in line with state-of-the-art methodology described in the literature, providing full transparency and reproducibility.

## 2. Materials and Methods

**Figure 1:**
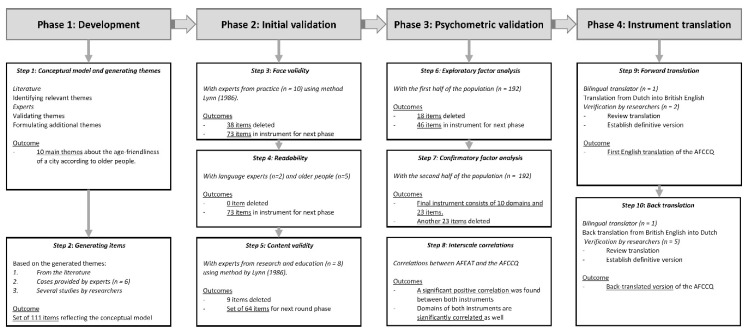
Flowchart representing the phases and steps for developing the Age-Friendly Cities and Communities Questionnaire (AFCCQ).

The Age-Friendly Cities and Communities Questionnaire (AFCCQ) for measuring the age-friendliness of a city was developed in a step-by-step approach. For the development and validation of the questionnaire, we based our methods in line with the criteria stated by the COnsensus-based Standards for selection of health Measurement INstruments (COSMIN) [27]. The COSMIN initiative aims to reach consensus about which measurement properties are considered to be important, their most adequate terms and definitions, and how they should be assessed in terms of study design and statistics [28]. The development consisted of the following four phases: (1) development of the conceptual model, themes and items; (2) initial (qualitative) validation; (3) psychometric validation and (4) translating the instrument from Dutch into British English (Figure 1).

### 2.1. Phase 1: Development of the Conceptual Model, Themes and Questions of the AFCCQ (steps 1 and 2)

Within the first step, the goal was to find a theoretical base for the conceptual model of the questionnaire [29] to assess how older people experience the age-friendliness of their city. Models and themes regarding age-friendliness of cities described in international books, guides and scientific articles published after 2007 were identified and discussed by the research team (J.D., R.F.M.v.d.H., W.H.v.S., J.v.H.). This research team was diverse and complementary in each other’s respective field of expertise. J.v.H., R.F.M.v.d.H. and W.H.v.S. have a background in housing/technology, social work/andragogy and political sciences, and were responsible for the content of the questionnaire. J.D. has a background in nursing and gerontology and was also responsible for the content of the questionnaire. In addition, he has extensive experience in developing and validating measurement instruments.

Consensus was reached, finding a theoretical basis described in the Global Age-Friendly Cities Guide by WHO [1], as the research team found this guide the best fit with the objective of the study. This guide published a model of age-friendly cities and communities, consisting of eight domains (i.e., themes). These domains are outdoor spaces and buildings; transportation; housing; social participation; respect and social inclusion; civic participation and employment; communication and information; and community support and health services.

Then, the goal was to select and formulate items based on the conceptual model [29], which was the outcome of step 1. The “Checklist of Essential Features of Age-Friendly Cities” published by the WHO [15] was used as a basis. This checklist contains a large number of features which are essential to an age-friendly city and is based on the results of the WHO Global Age-Friendly Cities project consultation in 33 cities in 22 countries. The checklist is a tool for a city’s self-assessment and a map for charting progress. The features of this checklist, therefore, formed the foundation of the items for this questionnaire, as well as the WHO documents from 2015 and 2018 [12,16]. The study by Marston and van Hoof [18] on the importance of technology in age-friendly cities (and their age-friendly ecosystem framework), which was further demonstrated for the context of the municipality of The Hague by van Hoof et al. [19], was used as the basis for additional questions on technology in the daily lives of older people. The research report by Bottenheft et al. [30] on The Hague as an age-friendly city was used to come up with additional questions that pertain to the Dutch context of city planning, housing, transport and the system of healthcare. Other efforts, such as the Liveability Index by the American Association of Retired Persons (AARP) [31], which consider many aspects of the (built) environment, were also touched upon. The Hong Kong Special Administrative Region Government stimulates active and healthy ageing by focusing on a multi-dimensional approach. The Hong Kong dimension of financial adequacy [32] was used to produce additional questions related to one’s financial situation and skills, as well as feeling financially secure. It also alludes to the notion of equity in health as addressed by WHO, which is defined as the absence of avoidable or remediable differences among groups of people, whether those groups are defined socially, economically, demographically, or geographically [33], and is one of the pillars of the core indicators for age-friendly cities presented by WHO [16].

Items were generated by R.F.M.v.d.H. and J.v.H., which were then provided with multiple rounds of feedback by the other researchers (J.D., W.H.v.S., L.M.T.H.-J.). Next, the items were discussed by the research team until consensus was reached on content and objective. The outcome of this step was a full set of items, which was a depiction of the eight themes as described by the WHO [1] and two additional themes (technology and financial situation).

### 2.2. Phase 2: Initial (Qualitative) Validation (Steps 3 to 5)

The aim of Phase 2 was to assess face validity, readability and content validity and conduct item reduction to establish a definitive selection of items which adequately represent the construct [29].

#### 2.2.1. Step 3: Face Validity—Participants and Measurement

Face validity was assessed using a quantification method [34,35]. A panel from the Dutch Province of South-Holland (*n* = 14 invited), with a diverse expertise on different domains of the WHO’s model of age-friendly cities and communities was contacted for participation. Participants who were willing to participate received an e-mail invitation to rate the relevance of the AFCCQ items regarding construct, study population, and purpose on a four-point Likert scale (1 = not relevant, 2 = somewhat relevant, 3 = quite relevant, 4 = highly relevant). The comprehensiveness was measured by asking the participants whether the items covered the entire construct measured.

#### 2.2.2. Step 4: Readability—Participants and Measurement

The readability was first examined by older people (the target population) (*n* = 10 invited). They scored all items which were not excluded in the face-validity round (Step 2.1) on language (i.e., difficulty in wording, interpretation of wording and sentences, length of sentences and construction) and understandability of the questions using a ten-point Likert scale (1 = I do not understand the question to 10 = I have no difficulty in understanding the question). Participants were asked to explain a grade below six (i.e., insufficient).

The final questionnaire was examined and improved by “*De Stadskamer*” of the Municipality of The Hague, which specializes in research on how civilians experience the service level of the municipality. This unit did secondary analyses of the wording level (including richness of vocabulary), sentence level (including number of subordinate clauses), and text level (cohesion and structure).

#### 2.2.3. Step 5: Content Validity—Participants and Measurement

The content validity was determined by the same quantification method as used in Step 3 [34,35]. In this round, a Dutch panel of experts in the field of age-friendly cities were contacted for participation (*n* = 13 invited). All experts were contacted based on holding a doctoral degree and having a track record in the field of gerontology research, specifically in relation to age-friendly cities. Experts were asked by e-mail to independently appraise the relevance of the items with respect to the construct, study population, and purpose on a four-point Likert scale (1 = not relevant, 2 = somewhat relevant, 3 = quite relevant, 4 = highly relevant). Comprehensiveness was again evaluated by asking the experts whether the items comprised the assumed construct and if they missed items or themes.

#### 2.2.4. Statistical Analyses in Phase 2

For the face and content validity of the studies, the Item Content Validity Index (I-CVI) was used, which is defined as the proportion of experts who rate the content as valid (relevance rating of 3 or 4) [34,35]. In both steps, this was calculated for each item. Lynn [34] and Polit et al. [35] found an item to be excellent when the I-CVI value was greater than 0.78.

For the face-validity round (Step 3), items were considered to be excellent when I-CVI ≥ 0.90. These items were retained for the readability (Step 4) and content validity (Step 5) rounds. Items on the threshold (I.CVI = 0.70 to 0.89) were individually assessed by the research team and with consensus were in- or excluded. Items with an I-CVI score of ≤ 0.69 were excluded.

The readability (Step 4) was assessed on a ten-point Likert scale. Individual items with a mean score of < 6 were discussed by the research team and changes in language were made. The readability for the total questionnaire was calculated by averaging all mean grades per item.

In Step 2.3, content validity was assessed, and items were rated excellent when the I-CVI value was greater than 0.78 [34,35]. Items on the threshold (I-CVI = 0.75, having eight raters) were individually assessed by the research team and included or excluded on the basis of consensus. For complete scale validation, all I-CVI values were averaged in order to calculate a Scale Content Validity Index (S-CVIave), for which a value greater than 0.90 is considered excellent [35]. Data of all steps were analysed using SPSS version 25.0 (IBM Corp., Armonk, NY, USA).

### 2.3. Phase 3: Psychometric Validation (Steps 6 to 8)

The aim of Phase 3 was to statistically assess the validity of the AFCCQ. Exploratory Factor Analysis (EFA) was used to identify the underlying factor structure of the AFCCQ [36]. One of the most important functions of EFA is that it allows for determining how well the items of a scale align with latent factors. In case there is no alignment, or when items are too identical, items can be removed. This improves the overall quality of the instrument. In order to test the factor structure that resulted from the EFA, a Confirmatory Factor Analysis (CFA) was conducted using a second dataset. In CFA, the researchers explicitly test the hypotheses about which items measure which latent factors, and provide more robust validity evidence of the fit of the tested model with the data (i.e., that a scale accurately measures what it purports). In order to investigate the validity of the AFCCQ, the dataset was split randomly in half (*n* = 192 for each half) and an EFA was conducted using the first half of the data and a CFA on the second half. This approach is used to investigate validity from a single survey administration, especially when the sample is large enough so that resulting subsets represent enough observations to run multiple rounds of factor analyses [36,37,38].

#### 2.3.1. Data Collection and Participants in Phase 2

For the data collection phase, a sample of community-dwelling older people (65 years and over) were recruited (Table 1). In January 2019, there were 539,040 inhabitants in the municipality of The Hague (https://denhaag.incijfers.nl/jive), of whom 78,073 were aged 65 and over (14.55% of the urban population). A total of 94.6% live independently, or 73,857 inhabitants. With a margin of error of 5% and a confidence level of 95%, this means a total of 383 respondents were needed. The inclusion criteria of the representative sample meant that—ideally—31.3% of the sample had to have a migrant background (according to the definitions of CBS—Statistics Netherlands). The largest group of migrants (47.7%) are migrants from Western countries, followed by people from Suriname (23.0%), as well as Morocco, Turkey, Aruba and the former Netherlands Antilles (Curaçao, Sint Maarten, Bonaire, Sint Eustatius and Saba), and other non-Western countries. The main focus was on recruiting people with a non-Western migration background. The ratio between males and females is 45%–55% in The Hague. Participants came from all boroughs of the city, and older people are not evenly spread across The Hague. A representative distribution across the age cohorts was sought (65–69 (31.0%); 70–74 (27.4%) and 75+ (41.6%)), as well as for the share of the population living in a home that is either rented or owned (58% were owned by the dweller, and 42% rented) [39]. We also recruited people who either lived alone or with a spouse.

The research was carried out by *aha! marktonderzoek en marketingadvies*, Groningen, The Netherlands among members of an existing *Ouderenpanel* database, and additionally recruited older citizens of the municipality of The Hague, between July and September 2020. Participants from the database had consented to their participation by being a part of the panel. Additional participants were asked to sign informed consent forms prior to filling out the questionnaire. Only those aged 65 years or over who lived in their own home and were able to communicate in Dutch were included.

#### 2.3.2. Step 6: Exploratory Factor Analysis

Before running the EFA, the correlations among all items were examined in order to determine if the items that should be related to one another were, in fact, related. Item variance and means were also examined. Ideally, one would like to see relatively high item variance (indicating a wide range of response patterns) and a mean closer to the centre of the scale range [37]. After exploring the data, an EFA was conducted.

Then, the number of latent factors were determined using scree plots, parallel analysis and the conceptual model. The scree plot is a subjective method that allows identification of the number of factors by observing the plot of eigenvalues as a function of the number of factors. The part of the plot with the elbow of the graph indicates the number of factors to be extracted [36]. Then, a parallel analysis was performed which examines eigenvalues in the sample data compared with randomly generated data to determine the number of factors. Triangulation of these sets of information guided decisions about the number of factors to extract.

After determining the number of factors, a maximum likelihood EFA was conducted using the oblimin rotation. Communalities and the loadings of each item were examined to identify those with low or cross-loadings. Specifically, the authors classified any item with a loading of magnitude 0.32 or less as low loading, indicating that less than 10% of the item variance was shared with a latent factor [40], and any item that had loading onto another factor half the magnitude of the main loading as a cross-loading [41]. Low or cross-loading items and items with low communalities (>0.40) were removed one at a time.

#### 2.3.3. Step 7: Confirmatory Factor Analysis

With this CFA, the factor structure resulting from the EFA was tested. First the variance to unity, allowing the factors to co-vary, which is a similar approach to using the promax rotation in the EFA, was set. In order to evaluate the fit of the model, multiple fit indices were considered. First of all, the normed χ^2^ was used, which is less sensitive to sample size than the χ^2^. Some researchers tolerate values as high as 5 as an adequate model fit [42], while others insist normed χ^2^ should be 2 or less, and less than 1.0 is a poor model fit. Shadfar and Malekmohammadi [43] stated that a value below 2 is preferred, but a value between 2 and 5 is considered to be acceptable. Furthermore, the robust Comparative Fit Index (CFI) and Tucker Lewis Index (TLI) were tested, both of which should be 0.9 or greater [44]. The root-mean squared residual (SRMR) should be less than 0.08 for good fit [45]. Finally, the root-mean square error of approximation (RMSEA) was tested, for which MacCallum et al. [46] suggested thresholds of 0.01 or less for excellent fit, 0.05 or less for good fit, and 0.08 for moderate fit, whereas Hu and Bentler [45] described values between 0.05 and 0.1 as a moderate fit. Then, internal consistency of the final model was evaluated using composite reliability which is preferred over Cronbach’s alpha with CFA. A composite reliability value of 0.70 was considered appropriate for reliability [47].

#### 2.3.4. Step 8: Interscale Correlation

During data collection, the items of the AFCCQ were supplemented by the ten core questions of the AFEAT (Age-Friendly Environment Assessment Tool) by Garner & Holland [26]. This is a validated questionnaire, and its questions needed to be answered using the same five-point Likert scale as with the AFCCQ (ranging from Strongly Disagree—Disagree—Neither Agree/Disagree—Agree—Strongly Agree). It was hypothesised that sum scores of both scales are highly positively correlated (sig. >0.001), specifically the corresponding latent factors measured by sub-scales of both instruments. The Pearson’s correlation coefficient was used to test this hypothesis.

### 2.4. Phase 4: Instrument Translation (Steps 9 and 10)

The final set of items was translated from Dutch into British English according to the procedure described by Brislin [48] and Maneesriwongul and Dixon [49]. As the first step, the forward translation, a bilingual translator (native Dutch speaker) translated the items into British English. This translation was verified by two bilingual researchers (J.D., J.v.H.), who reviewed the translation and established a definitive version. For the second step, the backward translation, an independent native Dutch speaking translator, who was an English language expert, translated the questions back into the Dutch language. This translator did not have access to the original items written in the Dutch language (i.e., a blind translation). Finally, the original Dutch version of the items were compared with the back-translated version by two researchers (J.D., J.v.H.). If necessary, modifications were made until agreement was reached. The other research team members (R.F.M.v.d.H., W.H.v.S., L.M.T.H.-J.) validated the agreed translated version. This led to the final version of the translation of the instrument.

## 3. Results

### 3.1. Phase 1: Development of the Conceptual Model, Themes and Questions of the AFCCQ (Steps 1 and 2)

A total of 111 items were developed for a total of ten domains. The domain Housing consisted of 11 items; social participation of 15 items; respect and social inclusion of 14 items; civic participation and employment of 9 items; communication and information of 8 items; community support and health services of 16 items; outdoor spaces and buildings of 16 items; transportation of 9 items; technology of 7 items; and financial situation of 6 items (see Table S1 for all developed items and reasons for item exclusion in the different steps).

### 3.2. Phase 2: Initial (Qualitative) Validation

#### 3.2.1. Step 3: Face Validity

A total of 10 respondents scored the AFCCQ on face validity. Respondents were all women and had a mean age of 39.7 (SD = 17.2). Two respondents had a bachelor’s degree, seven a master’s degree and one a doctoral degree. All had experience (mean 8.1 years, SD = 8.3) working for older people in their current area of practice (policy advisor government, researchers, consultants). Of the total of 111 initially developed items, 38 were excluded from the initial AFCCQ after assessment of face validity scores (Table S1). Multiple items were changed in language following the feedback of this group. Items of the AFCCQ were considered comprehensive and no suggestions for extension were made.

#### 3.2.2. Step 4: Readability

A total of five older people (two men and three women) and two language experts scored the AFCCQ on readability. The older people had a mean age of 74.4 (ranging from 69–81 years old). All respondents had a bachelor’s (*n* = 5) or master’s (*n* = 2) degree and experience with themes regarding age-friendly cities (ranging from 2–50 years). The readability of the AFCCQ was considered excellent with a mean of 8.9. Of the 73 remaining items, seven items (9.6%) scored between 7 and 7.9; 26 items (35.6%) scored between 8.0 and 8.9; and 40 items (54.8%) scored between 9.0 and 10.0. No changes in the AFCCQ were made after this study.

#### 3.2.3. Step 5: Content Validity

In total, eight (international) experts in age-friendly cities participated in this step (six Dutch, two Belgian (Flemish)). Five experts were female and three were male. Their mean age was 42 years (SD = 5.7). All held a doctoral degree and had ample experience within the field. Of the 73 remaining items, nine were excluded in this step after assessment of the content validity (Table S1). The S-CVIave of the remaining 64 items was 0.95 (range 0.88–1.00). Items of the AFCCQ were considered comprehensive, and no suggestions for extension were made.

### 3.3. Phase 3: Psychometric Validation

#### 3.3.1. Step 6: Exploratory Factor Analysis

The parallel analysis indicated five factors. However, the scree plot was fitting to the number of factors as expected from the conceptual model (10 factors). Therefore, we decided to continue with this number of factors. Then items with low communalities (<0.40) were removed (*n* = 8). The communality of an item represents how much variation of that item is explained by the latent factors. Although an item’s communality should ideally be 0.80 or greater, it is common for communalities to range between 0.40 and 0.70 [50]. Then, items with low (*n* = 1) or cross-loadings (*n* = 9) were removed one at a time. This iterative process left a set of 46 items with factor loadings that ranged from 0.35 to 0.88 (Table 2). Ten items loaded on another factor than initially thought when exploring the results, but all could be explained. For example, item 19 *“If necessary, I can get special community transport”* was originally included in the domain “Social participation” but loaded strongly on domain “Community support and health services”, which can be explained as the origin of the item can relate to both domains. Items with different factor loadings than originally thought were, therefore, replaced in a further step of the analysis as indicated by the EFA results.

#### 3.3.2. Step 7: Confirmatory Factor Analysis

The next step was to test the fit of the other half of the data with the structure determined by the EFA. This second step is used to confirm that the fit from the EFA is consistent in a more stringent and robust hypothesis-testing model in CFA. The resulting CFA model indicated that the ten-factor model of the AFCCQ did not fit the data well mainly due to low factor loadings. Therefore, we ran several models to maximise model fit with the data (Table 3). First, all items with factor loadings < 0.50 were excluded (model 2), followed by exclusion of all items with loadings < 0.60 (model 3) and excluding all items with loadings < 0.70 (model 4). Finally, items with problematic standardised residual covariances were excluded from the model (model 5). Of the final model, the value of the normed χ^2^ was 1.619, which indicates a good fit. Values of the robust CFI and the robust TLI were, respectively, 0.937 and 0.923, both above the 0.9 threshold [44]. The RMSEA was 0.057, which is lower than 0.08 (threshold for moderate fit [46]). The robust SRMR was 0.0569, which is below 0.08. This is considered to be a good fit according to Hu and Bentler [45].

**Figure 2:**
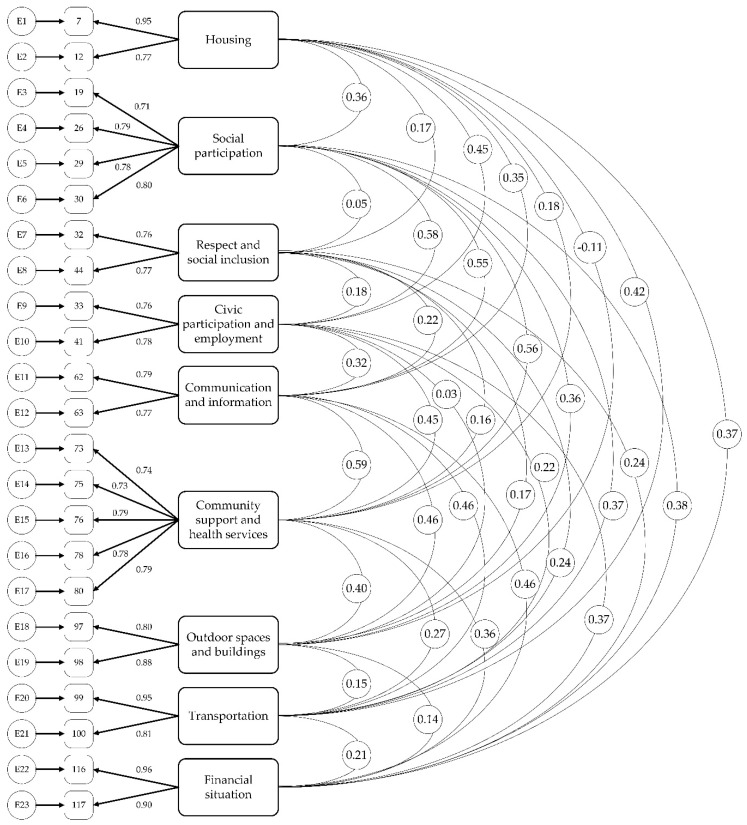
Model of the final confirmatory factor analysis.

Furthermore, the estimated covariance paths between the factors were all lower than the suggested 0.85 cut-off, indicating discriminant validity. Figure 2 shows the final model. Discriminant validity ensures that the items measure distinct, but perhaps related, factors. Overall, the results of the CFA suggested that the final model resulted from the EFA was (after trimming) a good fit on a second set of data.

Finally, the internal consistency of the model that emerged from the final CFA was examined by calculating the composite reliability per factor (Table 4). All factors demonstrate a value above the threshold for reasonable reliability of > 0.70 that is often reported. The thresholds for composite reliability are up for debate, with different authors offering different threshold suggestions. A lot depends upon how many items there are in the developed scale. Smaller numbers of scale items tend to result in lower reliability levels, while larger numbers of scale items tend to have higher level factors, with five to eight items that should meet a minimum threshold of 0.80 [51]. The results of the present study are in line with these findings.3.3.3. Step 8: Interscale Correlations

Table 5 presents the correlations between the AFCCQ and the AFEAT [26]. The hypothesis that the sum scores of both scales are highly positively correlated (r = 0.75, *p* < 0.01) was confirmed (Table 5). Furthermore, the hypothesis that corresponding domains of both instruments were also correlated was confirmed. These results provide evidence for good convergent validity of the AFCCQ.

### 3.4. Phase 4: Instrument Translation (Steps 9 and 10)

Overall, the forward translation was correctly performed by an independent, professional translator. However, some small changes were made by the main researchers to accomplish consequent use of British English (for instance, the word “plenty” was changed to “sufficient” or “enough” and “sick” was changed to “ill”). The end product of the back translation was rather similar to the original items. Occasionally, a loose translation was applied, and some word choices did not fit completely. Consensus was reached that no changes were necessary on the final version of the back translation. Because the aim of this phase was a good translation from Dutch into the British English language, cultural issues were not taken into further consideration. Both the Dutch and British English versions of the AFCCQ can be found in Table A1 and Table A2.

### 3.5. Interpretation and Presentation of Results

The AFCCQ can be used for both research and policy purposes. Total scores range from −46 to +46 points. The number of points per dimension can vary as the number of questions asked per domain varies, too. In order to simplify the interpretation of results and communicate with a larger community of stakeholders, it is advised to use a colour scheme principle (Table 6). As shown, there are several coloured zones. These zones represent how satisfied older people are regarding the city as a whole or a specific domain. Scores in the red zone mean people feel neutral to slightly unsatisfied (−) to very unsatisfied (−−−−). Light green (+) means people feel neutral to slightly satisfied. Scores in the darker green zones mean that people feel satisfied (++) to very satisfied (++++) with their respected city and/or a specific domain. This method allows for a clear and straightforward presentation of findings to a mixed audience, which is in line with the recommendations by Bucker et al. [13,14]. By doing so, policymakers can easily see in which domains they need to act. It can help in prioritising the domains and corresponding interventions for increasing the age-friendliness of their city, as long as the required interventions are a part of the tasks carried out by municipalities or city councils. The colour codes also indicate a sense of urgency. Red scores indicate the need for (immediate or necessary) action. Light green zones indicate that actions may still be needed, and darker green zones mean that there is still room for improvement. Over-time scores can be compared to assess whether policy decisions impacted the age-friendliness of the cities according to research participants. Researchers are advised to use the absolute scores in their analyses. Furthermore, cross-cultural validation may allow researchers to compare between cities within a given country, or between countries.

Table 6 presents the preliminary results of the sample used in this validation study. These results show how the scores can be presented and interpreted. The AFCCQ presents a relatively high item variance (indicating a wide range of response patterns) and a mean closer to the centre of the scale which is preferred. Furthermore, the results show that most domains score in the light green (++) category, meaning there’s room for improvement and actions are wanted. The domains of “Community support and health services” and “Outdoor spaces and buildings” score in the light green zone (+). This means that these two domains may be a priority for policy makers and social programmes or interventions and could be explored in further detail in order to increase the age-friendliness of the city as a whole. Zooming in at the neighbourhood level can further uncover (large) differences in AFCCQ scores and domains and pinpoint where to implement certain interventions.

## 4. Discussion

The step-by-step rigorous process of development and validation resulted in a valid, psychometrically sound, comprehensive 23-item questionnaire: The Age-Friendly Cities and Communities Questionnaire (AFCCQ) which is reported in full transparency. The AFCCQ can be used to measure the age-friendliness of a city or community. To date, such a validated tool was lacking, and many cities trying to assess their age-friendliness had to resort to a qualitative or mixed methodology approach, which was often based on the Checklist of Essential Features of Age-Friendly Cities [15]. The AFCCQ is the first validated tool that can be used for a quantitative assessment, which still allows for additional qualitative data to be shared with researchers or policy makers. The questions that were not included in the final instrument can nevertheless be used as a source of inspiration for a more in-depth survey per domain. The AFCCQ allows practitioners and researchers to capture the age-friendliness of a city or community in a numerical fashion, which helps to monitor the progress (or decline) of the age-friendliness and the potential impact of policies or social programs.

One of the main questions that needs to be resolved pertains to a cross-cultural validation. Is the current AFCCQ too Dutch in character? The Netherlands have a long-standing tradition of state-organized long-term care, with a nationwide approach to organized nursing home care and district nursing and home, social and domestic care taken care of by the municipalities. This means that for decades, the country has put great effort in improving the well-being of older citizens. This frontrunner position may impact the construct of the questionnaire and its constituting questions. In order to overcome this challenge, two international experts (Belgian nationality) were consulted who had a critical look at items that may have been too Dutch in character. This should have benefits for future cross-cultural validation procedures. Even though a rigorous translation process has been performed as a first step for future cross-cultural research, researchers who want to use the AFCCQ in their respective countries should test the cultural adaptation of the AFCCQ before using it to collect data, especially when cultures are very different from the Dutch/Western Europe culture. One of the most rigorous ways researchers can assess the cross-cultural validity of the AFCCQ is by the assessment of the measurement invariance (MI). MI assesses whether different groups respond in a similar way to a measurement instrument and its items [52,53]. Only when measurement instruments have a certain level of MI can average scores on (sub)scales between different countries/cultures be compared and meaningful interpretations of results be made. One challenge with this analysis is that data from both countries are needed [52]. 

A more user-friendly approach was presented by Sousa and Rojjanasrirat [54], who described a seven-step guideline from translation (step 1–4) pilot testing (step 5–6) to full psychometric testing (step 7). For the use of the AFCCQ in English-speaking countries, steps 1–4 were already performed in phase 4 of this study. For non-English speaking countries, these steps should be repeated from the translated British-English version into the language of choice. 

Some studies from other Western countries have addressed issues concerning national priorities. The study by Zaman and Thornton [25] from Australia identified the priority indicators for age-friendly development at the local government level in South Australia. In their words, the WHO’s age-friendly indicators are generalised and overarching and need modification by considering local needs. Garner and Holland [26] did similar important work from a British perspective. Their works show that a meticulous cross-cultural validation may be an important aspect in moving the AFCCQ further. At the same time, the AFCCQ was largely based on the Checklist of Essential Features of Age-Friendly Cities [15], which is also a first indication that most of the factors constituting the questionnaire are international in their origin and applicability.

In addition, Buffel et al. [55]—referring to the Checklist of Essential Features of Age-Friendly Cities [15]—raised the question whether the use of a universal checklist of action items is the most adequate method to deal with the diversity of cities and heterogeneity of their populations. Creating age-friendly communities will require an adjustment of methods and instruments to highly unequal local contexts. This applies not only to the diversity between but also within cities. The question can be asked to what extent instruments such as the AFCCQ survey do justice to the diversity of older people in the city, and whether indicators and items used reflect the different needs, concerns and preferences of particular groups of older people in the city and what they consider to be important aspects of an age-friendly city. This becomes even more important given the growing number of older people with a migration background [52], but also when we take into account existing forms of social inequality and deprivation [3,6]. This applies not only to ethnic or cultural diversity, but also to other factors such as gender, age, socioeconomic position or health status, which influence what people consider important when it comes to the age-friendliness of their living environment. Garner and Holland [26], for example, point to the relation between individual functioning and frailty and perceptions of environmental age-friendliness. When exploring the older people’s perception of their neighbourhood, Scharf et al. [56] found that older people’s ethnic background has an important influence on people’s perceptions. This highlights the importance of paying attention to how dimensions such as gender, ethnicity, income and material deprivation, educational level, household composition and health situation impact on the perception of the age-friendliness of the city and the immediate living environment. This requires that indicators and data can be disaggregated by such dimensions. At the same time, this is also in line with the WHO’s plea [16] to disaggregate data—from a perspective of equity—by social stratifications such as gender, age, ethnicity, socioeconomic status and neighbourhood. At the same time, these factors may influence the perceptions different groups have of the age-friendliness of their living environment. The novel AFCCQ provides a tool to move the plethora of smaller qualitative studies towards a more integrative approach of doing research, in which large-scale quantitative studies are supplemented by qualitative approaches. The AFCCQ allows for the inclusion of the abovementioned minority groups, such as people facing social inequalities, and their voices can be analysed separately in a quantitative manner. Quantitative data can help map the magnitude of social inequalities, also between subpopulations. A mixed-methods approach can help study the numerical data in more detail, for instance, when the AFCCQ domains are also studied through qualitative methodologies.

There may be a bias towards the ninth domain of financial situation (as it was taken from Hong Kong SAR (financial adequacy), and because all older people in The Netherlands enjoy a state pension under the 1956 General Old Age Pensions Act). This domain was not part of the original WHO model. Therefore, the panellists did not recognize these elements as age-friendly city indicators or a separate domain. The same can be said for the questions on technology, which correlated with other existing domains, and most questions (*n* = 5) were excluded in the qualitative initial validation rounds. The experts consulted in this study may not have recognized the importance of technology as an integral part of age-friendliness [8,57,58] yet. This may change in the future. At the same time, Marston and van Hoof [18] called for an integrated consideration of technology, and gerontechnology in particular, in all domains of age-friendly cities, instead of technology being a separate novel domain. Future studies could pay particular attention to the role of technology in the structure of the AFCCQ. When doing evaluation studies of age-friendly cities, technology should nevertheless be addressed explicitly, both qualitatively or using an additional instrument addressing the role of gerontechnology.

Local governments and city councils can use the AFCCQ to study the age-friendliness of their respective jurisdictions. The colour scheme approach helps communicate the results of such studies to a larger audience, including older citizens. One of the strengths of the AFCCQ is that it collects data among older people themselves instead of their representatives. Policy makers may even be encouraged to move up in the sequence of colours presented by the colour scheme as a motivator through their social and urban planning policies. Policy makers may also ask for additional research in the fields with low scores. Urban planners and architects could use the outcomes of the AFCCQ as a first indication of satisfaction with aspects of the built environment. At the same time, organisations for the interests of older citizens can use the AFCCQ as well in order to provide a foundation for their actions. Researchers can apply the AFCCQ as a quick scan of a particular city, or to measure age-friendliness in a longitudinal manner, following up on a cohort in a sequence of years. After going through the procedure of cross-cultural validation, cities in various countries (in and between various countries) could be compared as well.

Some considerations regarding this study should be discussed. First, considering the representativity and size of the sample used. The representativeness of the convenient sample of community-dwelling older people (60 years and over) can be questioned as selection bias (the panel used for the study, Dutch speaking older people living in their own home). This may have led to a bias as older people with strong positive or negative experiences/emotions are more likely to participate in such panels. However, for this study, this is acceptable as the primary focus was on the psychometric validation of the AFCCQ and not an exploration of experiences of Dutch older people living in the municipality of The Hague. Regarding the sample size, there is an abundance of recommendations for the appropriate sample size to use when conducting a factor analysis. Suggested minimum sample sizes range from three to 20 times the number of variables, and absolute ranges from 100 to over 1000 participants [59]. Even though this study met the minimum criterion of 1:3 (which means a minimum number of 135 participants), the sample size in this study is on the lower end of the advised number of participants. However, the sample size did not affect the performance of analysis, and over time, multiple studies have demonstrated that rather small sample sizes can be sufficient [60,61]. One strength in this study was the lack of missing data, which maximizes the validity of the item selection during the item reduction process. Now that the AFCCQ is considered psychometrically valid, imputation of data can be performed by researchers in future studies that focus on measuring the experiences and opinions of older people regarding the age-friendliness of their respected localities. Finally, the AFCCQ provided an answer to three of the recommendations made by Buckner et al. [14] regarding the challenges for evaluation of age-friendly city initiatives. The AFCCQ is a validated instrument which (i) can be applied in different contexts, (ii) can make assessments of the effectiveness of an intervention and (iii) presents findings clearly to a mixed audience.

## 5. Conclusions

The step-by-step rigorous process of development and validation resulted in a valid, psychometrically sound, comprehensive 23-item questionnaire: The Age-Friendly Cities and Communities Questionnaire (AFCCQ), which is reported in full transparency. The AFCCQ was derived through the COnsensus-based Standards for selection of health Measurement INstruments (COSMIN). The AFCCQ covers the eight domains of the WHO Age-Friendly Cities model, and an additional domain of financial situation. The AFCCQ allows practitioners and researchers to capture the age-friendliness of a city or community in a numerical fashion, which helps to monitor the progress (or decline) of the age-friendliness and the potential impact of policies or social programmes. Before the AFCCQ can be used in other countries and cities, it is encouraged to go through the process of cross-cultural validation. In order to facilitate the first steps of such a process, the AFCCQ was translated into British English. Therefore, the AFCCQ is now available in both Dutch and British English.

## Figures and Tables

**Table 1:** Demographics of participants (*n* = 384).

**Sex**	
Male	*n* = 188 (49%)
Female	*n* = 196 (51%)
**Age**	
Mean (SD)	74.4 (6.36)
60–65	*n* = 10 (2.6%)
65–69	*n* = 82 (21.4%)
70–74	*n* = 118 (30.7%)
75+	*n* = 156 (40.6%)
Missing values	*n* = 17 (4.7%)
Born in the European part of the Kingdom of the Netherlands (%) ^1^	*n* = 329 (85.7%)
**Educational level**	
Primary education only	*n*= 19 (4.9%)
Secondary school giving entry to intermediate vocational education	*n* = 101 (26.4%)
Intermediate vocational education	*n* = 64 (16.7%)
Secondary school giving entry to university (of applied sciences)	*n* = 35 (9.1%)
University of applied sciences	*n* = 95 (24.7%)
University	*n* = 70 (18.2%)
**Years living in The Hague**	
Mean (SD)	51.3 (22.7)
**Type of dwelling**	
Owner-occupant	*n* = 230 (59.9%)
Social housing	*n* = 110 (28.6%)
Private rent	*n* = 44 (11.5%)
**Living together with a partner** (%)	*n* = 216 (56.3%)
**Receiving care** (%)	*n* = 98 (25.5%)
**Living with one or more chronic conditions** (%)	*n* = 186 (48.4%)
**Using a wheeled walker or wheelchair** (%)	*n* = 56 (14.6%)

^1^ Denotes a possible migration background according to Dutch definitions.

**Table 2:** Item Communality and Final Exploratory Factor Analysis Results.

Item	Communality	Factor Loading									
		Housing	Social Participation	Respect and Social Inclusion	Civic Participation and Employment	Communication and Information	Community Support and Health Services	Outdoor Spaces and Buildings	Transportation	Technology	Financial Situation
1	0.702	0.30									**0.67**
3	0.621	**0.51**									
4	0.677	**0.76**									
9	0.628	**0.77**									
10	0.699	**0.41**									
12	0.611		**0.64**								
13	0.611		**0.56**								
16	0.632		**0.67**								
19	0.517						**0.62**				
20	0.505		**0.45**								
21	0.690		0.50						0.38		
22	0.676		**0.66**								
24	0.572										**0.41**
25	0.685		**0.76**								
26	0.639		**0.77**								
27	0.672			**0.74**							
39	0.632			**0.70**							
28	0.538				**0.58**						
36	0.542				**0.51**						
41	0.520				**0.54**						
42	0.495				0.45	0.32					
43	0.566			0.32	0.56						
47	0.625				0.36	0.39					
48	0.564				0.38		0.47				
50	0.506		**0.35**								
54	0.550					**0.54**					
55	0.542					**0.51**					
56	0.578					**0.58**					
57	0.565		0.31			0.51					
59	0.602								**0.49**		
61	0.596						**0.74**				
62	0.554						**0.54**				
64	0.567						**0.58**				
66	0.575						**0.67**				
67	0.637						**0.76**				
69	0.670						**0.54**				
71	0.568						**0.62**				
73	0.533										
74	0.661								**0.53**		
75	0.695								**0.52**		
76	0.544			**0.51**							
78	0.522		0.30						0.36		
83	0.547							**0.48**			
84	0.562							**0.40**			
85	0.644					0.59					
86	0.654					**0.52**			0.37		
88	0.601							**0.37**			
89	0.652							**0.40**			
90	0.711								**0.76**		
91	0.718								**0.82**		
92	0.663								**0.74**		
93	0.548								**0.40**		
96	0.619					0.35		0.32	0.46		
98	0.564							**0.70**			
106	0.815										**0.87**
107	0.827										**0.88**

Grey cells denote items that demonstrate no loading (item 83) or cross-loadings. bold indicate the included item and the corresponding domain.

**Table 3:** Models maximising model fit with the data.

Model	Normed χ^2^	Comparative Fit Index (CFI)	Tucker Lewis Index (TLI)	Root-Mean Squared Residual (SRMR)	Root-Mean Square Error of Approximation (RMSEA)
Model 1 (45 variables)	2.068	0.757	0.736	0.1041	0.075
Model 2 (35 variables) Exclusion of items with loadings below < 0.50	1.968	0.849	0.829	0.0820	0.071
Model 3 (31 variables) Exclusion of items with loadings < 0.60	1.960	0.878	0.857	0.0796	0.071
Model 4 (27 variables) Exclusion of items with loadings < 0.70	1.752	0.913	0.915	0.0628	0.063
Model 5 (23 variables)	1.619	0.937	0.923	0.0569	0.057

**Table 4:** Reliability per factor of the AFCCQ.

Domain	Housing	Social Participation	Respect and Social Inclusion	Civic Participation and Employment	Communication and Information	Community Support and Health Services	Outdoor Spaces and Buildings	Transportation	Financial Situation
**Composite Reliability**	0.85	0.85	0.74	0.74	0.76	0.88	0.83	0.88	0.93

**Table 5:** Interscale correlations (*r*) between Age-Friendly Environment Assessment Tool (AFEAT) by Garner & Holland [26] and the AFCCQ: total scale and sub-domains (*n* = 384).

Scales and Domains	AFEAT	AFEAT—Housing	AFEAT—Social Participation	AFEAT—Civic Participation and Employment	AFEAT—Communication and Information	AFEAT—Transportation
AFCCQ total	0.748 **					
AFCCQ—Housing	0.416 **	0.561 **	0.309 **	0.243 **	0.200 **	0.292 **
AFCCQ—Social participation	0.613 **	0.366 **	0.626 **	0.456 **	0.380 **	0.328 **
AFCCQ—Civic participation and employment	0.516 **	0.290 **	0.225 **	0.444	0.356 **	0.306 **
AFCCQ—Communication and information	0.480 **	0.310 **	0.373 **	0.375 **	0.456 **	0.347 **
AFCCQ—transportation	0.507 **	0.532 **	0.298 **	0.261 **	0.251 **	0.551 **

** Correlation is significant at the 0.01 level (2-tailed). Grey cells indicate the expected correlated domains of both scales.

**Table 6:** AFCCQ: Interpretation and presentation (*n* = 384).

Scale and Domains of the AFCCQ	Colour Scheme Principle								Mean	SD	Variance	Range
	**− − − −**	**− − −**	**− −**	**−**	**+**	**++**	**+++**	**++++**				
AFCCQ Total score	≤−35.1	−23.1–−35.0	−11.5–−23.0	−11.4–0.0	0.1–11.4	11.5–23.0	23.1–35.0	≥35.1	13.3	7.86	61.7	66
Housing	≤−3.1	−2.1–−3.0	−1.1–−2.0	−1.0–0.0	0.1–1.0	1.1–2.0	2.1–3.0	≥3.1	2.3	1.4	1.1	6
Social participation	≤−6.1	−4.1–−6.0	−2.1–−4.0	−2.0–0.0	0.1–2.0	2.1–4.0	4.1–6.0	≥6.1	2.5	1.0	5.8	8
Respect and social inclusion	≤−3.1	−2.1–−3.0	−1.1–−2.0	−1.0–0.0	0.1–1.0	1.1–2.0	2.1–3.0	≥3.1	1.6	1.5	2.5	8
Civic participation and employment	≤−3.1	−2.1–−3.0	−1.1–−2.0	−1.0–0.0	0.1–1.0	1.1–2.0	2.1–3.0	≥3.1	1.4	1.3	1.7	8
Communication and information	≤−3.1	−2.1–−3.0	−1.1–−2.0	−1.0–0.0	0.1–1.0	1.1–2.0	2.1–3.0	≥3.1	1.4	1.3	1.6	8
Community support and health services	≤−7.6	−5.1–−7.5	−2.6–−5.0	−2.5–0.0	0.1–2.5	2.6–5.0	5.1–7.5	≥7.6	2.5	2.9	8.2	20
Outdoor spaces and buildings	≤−3.1	−2.1–−3.0	−1.1–−2.0	−1.0–0.0	0.1–1.0	1.1–2.0	2.1–3.0	≥3.1	0.9	1.4	1.9	8
Transportation	≤−3.1	−2.1–−3.0	−1.1–−2.0	−1.0–0.0	0.1–1.0	1.1–2.0	2.1–3.0	≥3.1	1.7	1.5	2.2	8
Financial situation	≤−3.1	−2.1−3.0	−1.1–−2.0	−1.0–0.0	0.1–1.0	1.1–2.0	2.1–3.0	≥3.1	1.8	1.3	1.6	6

The coloured zones represent how dissatisfied or satisfied older people are regarding the city as a whole or a specific domain. Scores in the red zone mean people are neutral to slightly unsatisfied (−) to very unsatisfied (− − − −). Scores in the green zones mean that people are neutral to slightly satisfied (+) to very satisfied (++++).

## Supplementary Materials

The following are available online at https://www.mdpi.com/1660-4601/17/18/6867/s1. Table S1. Reasons for item exclusion in all steps for the Age-Friendly Cities and Communities Questionnaire (AFCCQ).

## Appendix A

**Table A1:** The Age-Friendly Cities and Communities Questionnaire (AFCCQ) in Dutch.

De Age Friendly Cities and Communities Questionnaire (AFCCQ)								
Alle Vragen van de AFCCQ Kunnen Beantwoord Worden op een 5-Punt Likert-Schaal van −2 (Helemaal Oneens); −1 (Oneens); 0 (Noch Mee Eens, Noch Mee Oneens); 1 (Eens); 2 (helemaal Eens).								
Item	Domein							
	**Huisvesting**							
Q1	Mijn woning is toegankelijk voor mij.							
Q2	Mijn woning is toegankelijk voor mensen die mij willen bezoeken.							
	**Sociale participatie**							
Q3	In mijn buurt zijn voldoende gelegenheden om mensen te ontmoeten.							
Q4	Activiteiten en evenementen worden georganiseerd op voor mij bereikbare plaatsen.							
Q5	De informatie over activiteiten en evenementen vind ik voldoende en ook geschikt voor mij.							
Q6	Ik vind het aanbod van evenementen en activiteiten voldoende afwisselend.							
	**Sociale inclusie**							
Q7 *	Ik krijg wel eens vervelende of negatieve opmerkingen vanwege mijn leeftijd.							
Q8 *	Ik krijg wel eens te maken met discriminatie vanwege mijn leeftijd.							
	Burgerparticipatie en werkgelegenheid							
Q9	Ik heb voldoende mogelijkheden om met jongere generaties om te gaan.							
Q10	Ik voel mij een gewaardeerd lid van de samenleving.							
	**Communicatie en informatie**							
Q11	Gedrukte en digitale informatie van de gemeente en andere maatschappelijke instanties zijn goed leesbaar qua lettertype en grootte.							
Q12	Gedrukte en digitale informatie van de gemeente en andere maatschappelijke instanties zijn geschreven in begrijpelijke taal.							
	**Sociale en gezondheidsvoorzieningen**							
Q13	Het aanbod van zorg en welzijn in mijn stad is voor mij voldoende.							
Q14	Als ik ziek ben, krijg ik de zorg en hulp die ik nodig heb.							
Q15	Indien nodig, kan ik zorg en welzijn telefonisch en fysiek gemakkelijk bereiken.							
Q16	Ik heb voldoende informatie over zorg en welzijn in mijn buurt.							
Q17	Zorg en welzijn werkers in mijn buurt zijn voldoende respectvol.							
	**Buitenruimte en gebouwen**							
Q18	Mijn buurt is voldoende toegankelijk voor rollator of rolstoel.							
Q19	De winkels in mijn buurt zijn voldoende toegankelijk met rollator of rolstoel.							
	**Transport**							
Q20	Ik kan gemakkelijk instappen in de bus of tram in mijn buurt.							
Q21	De bus- en tramhaltes in mijn buurt zijn gemakkelijk te bereiken en te gebruiken.							
	**Financiën**							
Q22	Mijn inkomen is voldoende om zonder problemen in mijn basisbehoeften te voorzien.							
Q23	Ik kan goed rondkomen met mijn inkomen.							
**Interpretatie AFCCQ totaalscore en specifieke domeinen**								
	− − − −	− − −	− −	−	+	+ +	+ + +	+ + + +
AFCCQ Totaal score	≤−35.1	−23.1–−35.0	−11.5–−23.0	−11.4–0.0	0.1–11.4	11.5–23.0	23.1–35.0	≥35.1
Huisvesting	≤−3.1	−2.1–−3.0	−1.1–−2.0	−1.0–0.0	0.1–1.0	1.1–2.0	2.1–3.0	≥3.1
Sociale participatie	≤−6.1	−4.1–−6.0	−2.1–−4.0	−2.0–0.0	0.1–2.0	2.1–4.0	4.1–6.0	≥6.1
Sociale inclusie	≤−3.1	−2.1–−3.0	−1.1–−2.0	−1.0–0.0	0.1–1.0	1.1–2.0	2.1–3.0	≥3.1
Burgerparticipatie en werkgelegenheid	≤−3.1	−2.1–−3.0	−1.1–−2.0	−1.0–0.0	0.1–1.0	1.1–2.0	2.1–3.0	≥3.1
Communicatie en informatie	≤−3.1	−2.1–−3.0	−1.1–−2.0	−1.0–0.0	0.1–1.0	1.1–2.0	2.1–3.0	≥3.1
Sociale en geszondheids-voorzieningen	≤−7.6	−5.1–−7.5	−2.6–−5.0	−2.5–0.0	0.1–2.5	2.6–5.0	5.1–7.5	≥7.6
Buitenruimte en gebouwen	≤−3.1	−2.1–−3.0	−1.1–−2.0	−1.0–0.0	0.1–1.0	1.1–2.0	2.1–3.0	≥3.1
Transport	≤−3.1	−2.1–−3.0	−1.1–−2.0	−1.0–0.0	0.1–1.0	1.1–2.0	2.1–3.0	≥3.1
Financiën	≤−3.1	−2.1–−3.0	−1.1–−2.0	−1.0–0.0	0.1–1.0	1.1–2.0	2.1–3.0	≥3.1

Scoresysteem: Items met een * moeten gehercodeerd worden in tegenovergestelde richting (−2 = 2, −1 = 1, 0 = 0, 1 = −1, 2 = −2). Tel alle scores van de AFCCQ op, om de AFCCQ totaalscore te berekenen. Tel alle scores van de specifieke domeinen op om de domeinspecifieke score te berekenen.

**Table A2:** The Age-Friendly Cities and Communities Questionnaire (AFCCQ) in English.

The Age Friendly Cities and Communities Questionnaire (AFCCQ)								
All Questions of the AFCCQ Can Be Answered on a 5-Point Likert-Scale Ranging from: −2 (Totally Disagree); −1 (Disagree); 0 (Neutral); 1 (Agree); 2 (Totally Agree).								
Item	Domain							
	**Housing**							
Q1	My house is accessible to me.							
Q2	My house is accessible to the people who come to visit me.							
	**Social participation**							
Q3	There are enough opportunities to meet people in my neighbourhood.							
Q4	Activities and events are organised in places that are accessible to me.							
Q5	The information about activities and events is enough for me and also suitable for me.							
Q6	I find the range of events and activities sufficiently varied.							
	**Respect and Social inclusion**							
Q7 *	I sometimes get annoying or negative remarks because of my age.							
Q8 *	I sometimes face discrimination because of my age.							
	Civic participation and employment							
Q9	I have enough opportunities to interact with younger generations.							
Q10	I feel like a valued member of society.							
	**Communication and information**							
Q11	Printed and digital information from the municipality and other social institutions is easy to read in terms of font and size.							
Q12	Printed and digital information from the municipality and other social institutions is written in understandable language.							
	**Community support and health services**							
Q13	The supply of care and welfare in my city is enough for me.							
Q14	When I am ill, I receive the care and help I need.							
Q15	If necessary, I can easily reach care and welfare services by telephone and in person.							
Q16	I have enough information about care and welfare services in my neighbourhood.							
Q17	Care and welfare workers in my neighbourhood are sufficiently respectful.							
	**Outdoor spaces and buildings**							
Q18	My neighbourhood is sufficiently accessible for a wheeled walker or wheelchair.							
Q19	The shops in my neighbourhood are sufficiently accessible with a wheeled walker or wheelchair.							
	**Transportation**							
Q20	I can easily get on the bus or tram in my neighbourhood.							
Q21	The bus and tram stops in my neighbourhood are easy to reach and use.							
	**Financial situation**							
Q22	My income is sufficient to cover my basic needs without any problems.							
Q23	I live well on my income.							
**Interpretation AFCCQ total score and separate domains.**								
	− − − −	− − −	− −	−	+	+ +	+ + +	+ + + +
AFCCQ Total score	≤−35.1	−23.1–−35.0	−11.5–−23.0	−11.4–0.0	0.1–11.4	11.5–23.0	23.1–35.0	≥35.1
Housing	≤−3.1	−2.1–−3.0	−1.1–−2.0	−1.0–0.0	0.1–1.0	1.1–2.0	2.1–3.0	≥3.1
Social participation	≤−6.1	−4.1–−6.0	−2.1–−4.0	−2.0–0.0	0.1–2.0	2.1–4.0	4.1–6.0	≥6.1
Respect and social inclusion	≤−3.1	−2.1–−3.0	−1.1–−2.0	−1.0–0.0	0.1–1.0	1.1–2.0	2.1–3.0	≥3.1
Civic participation and employment	≤−3.1	−2.1–−3.0	−1.1–−2.0	−1.0–0.0	0.1–1.0	1.1–2.0	2.1–3.0	≥3.1
Communication and information	≤−3.1	−2.1–−3.0	−1.1–−2.0	−1.0–0.0	0.1–1.0	1.1–2.0	2.1–3.0	≥3.1
Community support and health services	≤−7.6	−5.1–−7.5	−2.6–−5.0	−2.5–0.0	0.1–2.5	2.6–5.0	5.1–7.5	≥7.6
Outdoor spaces and buildings	≤−3.1	−2.1–−3.0	−1.1–−2.0	−1.0–0.0	0.1–1.0	1.1–2.0	2.1–3.0	≥3.1
Transportation	≤−3.1	−2.1–−3.0	−1.1–−2.0	−1.0–0.0	0.1–1.0	1.1–2.0	2.1–3.0	≥3.1
Financial situation	≤−3.1	−2.1–−3.0	−1.1–−2.0	−1.0–0.0	0.1–1.0	1.1–2.0	2.1–3.0	≥3.1

Scoring system: Items with * should be recorded in the opposite direction (−2 = 2, −1 = 1, 0 = 0, 1 = −1, 2 = −2). Sum all scores of the AFCCQ for the AFCCQ total score. Sum all scores of separate domains for the domain specific score.
