# Supplementary material for: How Older People Experience the Age-Friendliness of Their City: Development of the Age-Friendly Cities and Communities Questionnaire
Source: Int J Environ Res Public Health. 2020 Sep 20;17(18):6867. doi: 10.3390/ijerph17186867 (PMC7559304; doi:10.3390/ijerph17186867)
Supplement: Supplementary file 1 [file ijerph-17-06867-s001.pdf]

International Journal of
Environmental Research and Public Health

MDPI

Article

How Older People Experience the Age-Friendliness of Their City: Development of the Age-Friendly Cities and Communities Questionnaire

Jeroen Dikken [1,2], Rudy F.M. van den Hoven [1], Willeke H. van Staalduinen [3], Loes M.T. Hulsebosch-Janssen [4] and Joost van Hoof [1,5,*]

1 Chair of Urban Ageing, Faculty of Social Work & Education, The Hague University of Applied Sciences, Johanna Westerdijkplein 75, 2521 EN Den Haag, Netherlands; J.Dikken@hhs.nl (J.D.); r.f.m.vandenhoven@hhs.nl (R.F.M.v.d.H.)
2 Faculty of Health, Nutrition & Sport, The Hague University of Applied Sciences, Johanna Westerdijkplein 75, 2521 EN Den Haag, Netherlands
3 AFEdemy—Academy on age-friendly environments in Europe, Buurtje 2, 2802 BE Gouda, Netherlands; willeke@afedemy.eu
4 Hulsebosch Advies, Lissenvaart 43, 2724 SJ Zoetermeer, Netherlands; hulsebosch@hm-advies.nl
5 Institute of Spatial Management, Faculty of Environmental Engineering and Geodesy, Wrocław University of Environmental and Life Sciences, ul. Grunwaldzka 55, 50-357 Wrocław, Poland
* Correspondence: j.vanhoof@hhs.nl; Tel.: +31-6-23381404

Received: 5 September 2020; Accepted: 16 September 2020; Published: date

Supplementary Table S1. Reasons for item exclusion in all steps for the Age-Friendly Cities and Communities Questionnaire (AFCCQ).

Item Nr.	Item Text	Step 3 I-CVI [a]	Step 4 Readability	Step 5 I-CVI [b]	Step 6 EFA	Step 7 CFA	Reason for Exclusion
	**Domain 1: Housing**						
1	I can pay my living expenses (rent/mortgage) without any problem.	1.0	8.7	1.0	0.67	<0.70	Low loading CFA
2	My house is well maintained.	0.7	-	-	-	-	Exclusion I-CVI consensus
3	My house has the facilities and adaptations that I need.	1.0	8.6	1.0	0.51	<0.60	Low loading CFA
**4**	**My house is accessible to me.**	**0.9**	**8.3**	**1.0**	**0.76**	**0.95**	**Included**
5	I can pay water, electricity, gas and municipal tax.	0.8	-	-	-	-	Exclusion I-CVI consensus
6	I believe my city has sufficient affordable and suitable homes for older people.	1.0	9.3	0.75	-	-	Exclusion I-CVI consensus
7	There are sufficient lifetime homes in my city.	0.6	-	-	-	-	Exclusion I-CVI score
8	If necessary, it is possible and affordable to have the necessary adjustments made to my home.	0.8	-	-	-	-	Exclusion I-CVI consensus
**9**	**My house is accessible to the people who come to visit me.**	**0.9**	**9.3**	**1.0**	**0.77**	**0.77**	**Included**
10	I feel safe and secure in my neighbourhood.	1.0	9.9	1.0	0.41	<0.70	Low loading CFA
11	My house in easy to keep clean.	0.8	-	-	-	-	Exclusion I-CVI consensus
	**Domain 2: Social Participation**						
12	For me, there are enough social, cultural or educational activities or other forms of leisure in my neighbourhood.	1.0	8.6	1.0	0.64	x	Problematic standardised residual covariances
13	There are enough sports activities available to me (alone or in a group) in my city.	0.9	8.6	1.0	0.56	x	Problematic standardised residual covariances

14	For me, there are plenty of opportunities in the city to participate in religious activities.	0.8	-	-	-	-	Exclusion I-CVI consensus
15	There are plenty of interesting activities in my neighbourhood.	0.9	8.9	0.75	-	-	Exclusion I-CVI consensus
**16**	**There are enough opportunities to meet people in my neighbourhood.**	**0.9**	**9.1**	**1.0**	**0.67**	**0.71**	**Included**
17	My neighborhood offers enough activities to participate in.	0.8	-	-	-	-	Exclusion I-CVI consensus
18	The number of times that I see or speak to friends or family a week is sufficient.	0.9	9.7	1.0	x	-	Communalities < 40
19	If necessary, I can get special community transport. *(Moved to the domain of Community support and health services)*	0.8	8.9	1.0	0.62	<0.50	Low loading CFA
20	The relationship/contact between the people in the neighbourhood is good.	0.9	8.0	0.88	0.45	<0.50	Low loading CFA
21	Amenities in my neighbourhood are easily accessible.	1.0	9.4	0.88	x	-	Cross-loading EFA
**22**	**Activities and events are organised in places that are accessible to me.**	**1.0**	**9.1**	**0.88**	**0.66**	**0.79**	**Included**
23	Activities and events are organised at convenient times for me.	0.7	-	-	-	-	Exclusion I-CVI consensus
24	Activities and events are affordable for me. *(Moved to the domain of Financial situation)*	0.9	9.1	1.0	0.41	<0.70	Low loading CFA
**25**	**The information about activities and events is enough for me and also suitable for me.**	**1.0**	**9.0**	**0.88**	**0.76**	**0.78**	**Included**
**26**	**I find the range of events and activities sufficiently varied.**	**0.9**	**9.1**	**0.88**	**0.77**	**0.80**	**Included**
	**Domain 3: Respect and Social Inclusion**						
**27**	**I sometimes get annoying or negative remarks because of my age.**	**1.0**	**9.9**	**0.88**	**0.74**	**0.76**	**Included**
**28**	**I have enough opportunities to interact with younger generations.** ***(Moved to the domain of Civic participation and employment)***	**1.0**	**9.3**	**0.88**	**0.58**	**0.76**	**Included**
29	Sufficient activities are being undertaken to improve the neighborhood.	0.4	-	-	-	-	Exclusion I-CVI score
30	I regularly associate with older people with a different background.	0.7	-	-	-	-	Exclusion I-CVI consensus
31	I believe that companies in my area and the government have tailored their services well to my needs.	0.9	8.6	0.75	-	-	Exclusion I-CVI consensus
32	I believe that staff working in the stores in my area take my needs into account.	0.8	8.9	0.5	-	-	Exclusion I-CVI consensus
33	I believe that the media in my area present a realistic picture of older people.	0.9	7.6	0.75	-	-	Exclusion I-CVI consensus
34	I believe that the posters and leaflets give a realistic image of older people.	0.7	-	-	-	-	Exclusion I-CVI consensus
35	Older people are sufficiently involved in educational activities.	0.7	-	-	-	-	Exclusion I-CVI consensus
**36**	**I feel like a valued member of society.** ***(Moved to the domain of Civic participation and employment)***	**1.0**	**9.0**	**1.0**	**0.51**	**0.78**	**Included**
37	Services (municipality, healthcare, welfare) are sufficiently accessible to people who are less well off financially.	0.8	-	-	-	-	Exclusion I-CVI consensus
38	Sometimes I am confronted with false imaging of older people.	0.7	-	-	-	-	Exclusion I-CVI consensus
**39**	**I sometimes face discrimination because of my age.**	**1.0**	**9.4**	**1.0**	**0.70**	**0.77**	**Included**
40	In case of age discrimination, I know where to go for help or to file a complaint.	0.9	8.7	1.0	x	-	Communalities < 40
	**Domain 4: Civic Participation and Employment**						

41	If I would like to, there are enough opportunities to do volunteer work that suits my interests and abilities.	1.0	9.4	1.0	0.54	<0.60	Low loading CFA
42	I feel sufficiently listened to in participation and decision making.	0.9	7.0	0.88	x	-	Cross-loading EFA
43	I am sufficiently involved in things that are going on in my neighbourhood.	0.9	9.3	0.88	x	-	Cross-loading EFA
44	I feel that municipal plans ABOUT older people have also been decided WITH older people.	1.0	7.1	0.88	x	-	Communalities <40
45	I can give my opinion on the services and facilities that are of interest to me.	0.8	-	-	-	-	Exclusion I-CVI consensus
46	I am involved in developments in the neighbourhood.	0.8	-	-	-	-	Exclusion I-CVI consensus
47	I am well informed about what is going on in the neighbourhood.	0.9	8.7	1.0	x	-	Cross-loading EFA
48	I do volunteer work on a regular basis.	0.9	9.6	0.88	x	-	Cross-loading EFA
49	In the past month I have been active as a volunteer at least once.	0.7	-	-	-	-	Exclusion I-CVI consensus
	**Domain 5: Communication and Information**						
50	There is enough information available about activities for seniors in my neighbourhood. *(Moved to the domain of Social participation)*	0.9	9.1	1.0	0.35	x	Problematic standardised residual covariances
51	There is sufficient municipal and neighborhood information available for older people.	0.6	-	-	-	-	Exclusion I-CVI score
52	There is sufficient word of mouth regarding activities in the neighborhood.	0.6	-	-	-	-	Exclusion I-CVI score
53	There is sufficient word of mouth regarding activities in the neighborhood for people who are socially isolated.	0.5	-	-	-	-	Exclusion I-CVI score
**54**	**Printed and digital information from the municipality and other social institutions is easy to read in terms of font and size.**	**1.0**	**8.7**	**0.88**	**0.54**	**0.79**	**Included**
**55**	**Printed and digital information from the municipality and other social institutions is written in understandable language.**	**0.9**	**8.7**	**1.0**	**0.51**	**0.77**	**Included**
56	When I call the municipality or another social institution I am well answered.	0.9	9.0	1.0	0.58	<0.50	Low loading CFA
57	If I have a complaint, I feel that I am listened to.	0.9	9.0	0.88	x	-	Cross-loading EFA
	**Domain 6: Community Support and Health Services**						
58	I think the hospitals in my city are senior-friendly.	0.6	-	-	-	-	Exclusion I-CVI score
59	The quality of care in my city is sufficient. *(Moved to the domain of Transportation)*	0.9	9.0	0.88	0.49	<0.50	Low loading CFA
60	I don't have to go far from home to visit a care service.	0.8	-	-	-	-	Exclusion I-CVI consensus
61	If I provide family care, I receive enough support.	1.0	9.0	0.88	0.74	<0.50	Low loading CFA
62	I receive sufficient domestic help at home.	0.9	8.6	1.0	0.54	<0.50	Low loading CFA
63	I can afford my healthcare costs.	0.8	-	-	-	-	Exclusion I-CVI consensus
**64**	**The supply of care and welfare in my city is enough for me.**	**1.0**	**9.0**	**0.88**	**0.58**	**0.74**	**Included**
65	The supply of care and welfare in my city is generally sufficient.	0.6	-	-	-	-	Exclusion I-CVI score
**66**	**When I am ill, I receive the care and help I need.**	**0.9**	**9.3**	**1**	**0.67**	**0.73**	**Included**
**67**	**If necessary, I can easily reach care and welfare services by telephone and in person.**	**0.9**	**7.7**	**1.0**	**0.76**	**0.79**	**Included**
68	If I want to visit a nursing home, I can easily go there.	0.7	-	-	-	-	Exclusion I-CVI consensus

**69**	**I have enough information about care and welfare services in my neighbourhood.**	**1.0**	**8.7**	**1.0**	**0.54**	**0.79**	**Included**
70	Healthcare in my city is sufficiently coordinated and easy to get.	0.7	-	-	-	-	Exclusion I-CVI consensus
**71**	**Care and welfare workers in my neighbourhood are sufficiently respectful.**	**0.8**	**8.7**	**0.88**	**0.62**	**0.78**	**Included**
72	The care and welfare facilities I need are affordable for me.	0.8	-	-	-	-	Exclusion I-CVI consensus
73	Communication about crisis situations in my city has consideration for seniors.	1.0	7.6	0.88	x	-	No Loading
			**Domain 7: Outdoor Spaces and Buildings**				
74	I can easily walk from A to B in my neighbourhood.	0.8	9.7	1.0	0.53	<0.60	Low loading CFA
75	Public buildings in my city are easily accessible to me.	1.0	9.4	1.0	0.52	<0.70	Low loading CFA
76	My neighbourhood and street are safe and secure. *(Moved to the domain of Respect and Social inclusion)*	0.9	7.6	1.0	0.51	<0.50	Low loading CFA
77	I can always park my car close to home. *(Moved to the domain of Transportation)*	0.8	8.4	0.88	x	-	Communalities < 40
78	Shops are close to my house.	0.9	8.6	0.88	x	-	Cross-loading EFA
79	Tiles in sidewalks are straight.	0.8	-	-	-	-	Exclusion I-CVI consensus
80	In my neighborhood, cycling and walking paths are well separated from each other.	0.9	9.6	0.75	-	-	Exclusion I-CVI consensus
81	There are parks near my home where I can go.	0.7	-	-	-	-	Exclusion I-CVI consensus
82	There are enough seats and benches in the parks and shopping malls in my neighbourhood.	1.0	9.0	1.0	x	-	Communalities < 40
83	There are enough clean and accessible public toilets in my neighbourhood.	1.0	8.3	1.0	0.48	<0.50	Low loading CFA
84	The public space in my neighbourhood (park, street, square) is clean and pleasant to be in.	1.0	9.6	1.0	0.40	<0.50	Low loading CFA
85	Pedestrian crossings in my neighbourhood are safe. *(Moved to the domain of Communication and Information)*	0.9	9.6	0.88	0.59	<0.60	Low loading CFA
86	I have enough time to cross at pedestrian crossings in my neighbourhood.	1.0	9.6	0.88	x	-	Cross-loading EFA
87	My city is sufficiently accessible with a walker or wheelchair.	0.8	-	-	-	-	Exclusion I-CVI consensus
**88**	**My neighbourhood is sufficiently accessible for a wheeled walker or wheelchair.**	**1.0**	**8.9**	**1.0**	**0.37**	**0.80**	**Included**
**89**	**The shops in my neighbourhood are sufficiently accessible with a wheeled walker or wheelchair.**	**1.0**	**9.6**	**1.0**	**0.40**	**0.88**	**Included**
			**Domain 8: Transportation**				
**90**	**I can easily get on the bus or tram in my neighbourhood.**	**0.9**	**9.7**	**0.88**	**0.76**	**0.81**	**Included**
**91**	**The bus and tram stops in my neighbourhood are easy to reach and use.**	**0.9**	**9.3**	**1.0**	**0.82**	**0.95**	**Included**
92	Public transportation in my city fits my needs.	0.9	9.3	0.88	0.74	x	Problematic standardised residual covariances
93	Public transportation in my city is affordable for me.	0.7	9.1	0.88	0.40	<0.50	Low loading CFA
94	Streets are safe enough for older people.	0.7	-	-	-	-	Exclusion I-CVI consensus
95	Public transport services are frequent.	0.7	-	-	-	-	Exclusion I-CVI consensus
96	The bus and tram drivers in my city are considerate of me.	0.9	9.1	0.88	x	-	Cross-loading EFA

97	The quality and availability of the community transport in my city (The Hague) is sufficient.	0.8	-	-	-	-	Exclusion I-CVI consensus
98	There are sufficient disabled parking spaces in my city. *(Moved to the domain of Outdoor spaces and buildings)*	0.9	8.3	0.88	0.70	<0.50	Low loading CFA
	**Domain 9: Technology**						
99	If I could contact my doctor electronically, I would.	0.8	-	-	-	-	Exclusion I-CVI consensus
100	If I could notify home care via an emergency response system, I would.	0.9	7.7	0.75	-	-	Exclusion I-CVI consensus
101	I would be happy if the doctor could check on me remotely and see me in a digital consultation.	1.0	8.1	1.0	x	-	Communalities < 40
102	I think technology supports and simplifies my life.	1.0	9.0	0.62	-	-	Exclusion I-CVI consensus
103	I am able to understand and use the digital services provided by the municipality, bank and public transport.	1.0	8.9	1.0	x	-	Communalities < 40
104	I am sufficiently adept at digital care and e-health.	0.7	-	-	-	-	Exclusion I-CVI consensus
105	I experience obstacles in my daily life due to technology.	0.9	8.9	0.75	-	-	Exclusion I-CVI consensus
	**Domain 10: Financial Situation**						
**106**	**My income is sufficient to cover my basic needs without any problems.**	**1.0**	**9.4**	**1.0**	**0.87**	**0.96**	**Included**
**107**	**I live well on my income.**	**0.9**	**9.9**	**1.0**	**0.88**	**0.90**	**Included**
108	I have appealed to others for financial support one or more times in the past 12 months.	0.8	-	-	-	-	Exclusion I-CVI consensus
109	I am concerned about my financial situation.	0.7	-	-	-	-	Exclusion I-CVI consensus
110	I know where to go with questions about money, for example at the municipality.	0.8	-	-	-	-	Exclusion I-CVI consensus

Item Content Validity Index (I-CVI)—number of experts rating a 3 or 4/total number of experts. [a]—Items with I-CVI ≤ 0.69 were excluded, 0.69 < I-CVI < 0.90 were individually assessed, I-CVI ≥ 0.90 were retained [b]—Items with I-CVI ≤ 0.78 were retained. AFCCQ average Scale-Content Validity Index (S-CVI*ave*) after step 3 = 0.95 (64 items). AFCCQ average Scale-Content Validity Index of the final instrument = 0.95 (23 items).
Bold items are included in the final AFCCQ. The grey cells indicate that items were excluded in the next step of the validation process. The letter x represents a non-numeric exclusion for next step.
